# Randomized Evaluation of Surgery in Elderly with Traumatic Acute SubDural Hematoma (RESET-ASDH trial): study protocol for a pragmatic randomized controlled trial with multicenter parallel group design

**DOI:** 10.1186/s13063-022-06184-1

**Published:** 2022-03-29

**Authors:** Ranjit D. Singh, Jeroen T. J. M. van Dijck, Thomas A. van Essen, Hester F. Lingsma, Suzanne S. Polinder, Erwin J. O. Kompanje, Erik W. van Zwet, Ewout W. Steyerberg, Godard C. W. de Ruiter, Bart Depreitere, Wilco C. Peul

**Affiliations:** 1 University Neurosurgical Center Holland, LUMC, HMC and Haga Teaching Hospital, Leiden and The Hague, J11 Albinusdreef 2, 2333 ZA Leiden, The Netherlands; 2 Centre for Medical Decision Making, Department of Public Health, Erasmus MC-University Medical Centre Rotterdam, Rotterdam, The Netherlands; 3 Department of Public Health, Erasmus MC-University Medical Centre Rotterdam, Rotterdam, The Netherlands; 4 Department of Intensive Care, Erasmus MC-University Medical Centre Rotterdam, Rotterdam, The Netherlands; 5 Department of Biomedical Data Sciences, Leiden University Medical Center, Leiden, The Netherlands; 6 Neurosurgery, University Hospital Leuven, Leuven, Belgium

**Keywords:** Traumatic brain injury, Neurosurgery, Acute subdural hematoma, Neurotrauma, Elderly, Randomized controlled trial, Pragmatic

## Abstract

**Background:**

The rapidly increasing number of elderly (≥ 65 years old) with TBI is accompanied by substantial medical and economic consequences. An ASDH is the most common injury in elderly with TBI and the surgical versus conservative treatment of this patient group remains an important clinical dilemma. Current BTF guidelines are not based on high-quality evidence and compliance is low, allowing for large international treatment variation. The RESET-ASDH trial is an international multicenter RCT on the (cost-)effectiveness of early neurosurgical hematoma evacuation versus initial conservative treatment in elderly with a t-ASDH

**Methods:**

In total, 300 patients will be recruited from 17 Belgian and Dutch trauma centers. Patients ≥ 65 years with at first presentation a GCS ≥ 9 and a t-ASDH > 10 mm or a t-ASDH < 10 mm and a midline shift > 5 mm, or a GCS < 9 with a traumatic ASDH < 10 mm and a midline shift < 5 mm without extracranial explanation for the comatose state, for whom clinical equipoise exists will be randomized to early surgical hematoma evacuation or initial conservative management with the possibility of delayed secondary surgery. When possible, patients or their legal representatives will be asked for consent before inclusion. When obtaining patient or proxy consent is impossible within the therapeutic time window, patients are enrolled using the deferred consent procedure. Medical-ethical approval was obtained in the Netherlands and Belgium. The choice of neurosurgical techniques will be left to the discretion of the neurosurgeon. Patients will be analyzed according to an intention-to-treat design. The primary endpoint will be functional outcome on the GOS-E after 1 year. Patient recruitment starts in 2022 with the exact timing depending on the current COVID-19 crisis and is expected to end in 2024.

**Discussion:**

The study results will be implemented after publication and presented on international conferences. Depending on the trial results, the current Brain Trauma Foundation guidelines will either be substantiated by high-quality evidence or will have to be altered.

**Trial registration:**

Nederlands Trial Register (NTR), Trial NL9012. ClinicalTrials.gov, Trial NCT04648436.

## Administrative information

Note: the numbers in curly brackets in this protocol refer to SPIRIT checklist item numbers. The order of the items has been modified to group similar items (see http://www.equator-network.org/reporting-guidelines/spirit-2013-statement-defining-standard-protocol-items-for-clinical-trials/).

**Table:** 

Title {1}	Randomized Evaluation of Surgery in Elderly with Traumatic Acute SubDural Hematoma (RESET-ASDH trial): study protocol for a pragmatic randomized controlled trial with multicenter parallel group design **Subtitle** Survival and quality of life after early surgical intervention versus wait-and-see in elderly patients with a traumatic acute subdural hematoma (ASDH).
Trial registration {2a and 2b}.	Nederlands Trial Register (NTR), Trial NL9012. ClinicalTrials.gov, Trial NCT04648436.
Protocol version {3}	Version 6.0, 22-12-2021
Funding {4}	This work is supported by the BeNeFIT grant (ZonMw & KCE) number 852101065. The Belgian Health Care Knowledge Centre (KCE) and ZonMw, the Netherlands Organisation for Health Research and Care Innovation launched a joint program under the name ‘BeNeFIT’. Extensive external peer review of the protocol was part of the multiple-round funding process. ZonMw: https://www.zonmw.nl E-mail: benefit@zonmw.nl KCE: https://kce.gov.be E-mail: trials@kce.fgov.be
Author details {5a}	Ranjit D. Singh^1^ Jeroen T.J.M. van Dijck*^1^ Thomas A. van Essen*^1^ Hester F. Lingsma^2^ Suzanne S. Polinder^3^ Erwin J.O. Kompanje^4^ Erik W. van Zwet^5^ Ewout W. Steyerberg^5^ Godard C.W. de Ruiter^1^ Bart Depreitere^6^ Wilco C. Peul^1^ *These authors contributed equally to this manuscript ^1^ University Neurosurgical Center Holland, LUMC, HMC & Haga Teaching Hospital, Leiden & The Hague, The Netherlands ^2^ Centre for Medical Decision Making, Department of Public Health, Erasmus MC-University Medical Centre Rotterdam, Rotterdam, The Netherlands ^3^ Department of Public Health, Erasmus MC-University Medical Centre Rotterdam, Rotterdam, The Netherlands ^4^ Department of Intensive Care, Erasmus MC-University Medical Centre Rotterdam, Rotterdam, The Netherlands ^5^ Department of Biomedical Data Sciences, Leiden University Medical Center, Leiden, The Netherlands ^6^ Neurosurgery, University Hospital Leuven, Leuven, Belgium **Corresponding author** Ranjit Dhillon Singh Postal address: Albinusdreef 2, 2333 ZA Leiden | J11 E-mail: r.d.singh@lumc.nl
Name and contact information for the trial sponsor {5b}	Leiden University Medical Center (LUMC), Dept. of Neurosurgery Wilco C. Peul, MD, MPH, PhD, MBa Professor and Chair Neurosurgery E-mail: w.c.peul@lumc.nl Tel. + 31715262109 Tel. Mobile: + 31651508781 Legally represented by: drs. Egbert Vos, Managing Director Division 3, LUMC, e.j.vos@lumc.nl
Role of sponsor {5c}	The funding agencies (ZonMw/KCE) have been actively involved in the recruitment planning and feasibility assessment of the trial. The medical and practical aspects of the study, including the decision to submit the protocol and final report for publication, lie completely with the sponsor (LUMC) together with the Belgian coordinating center (UZ Leuven). The first publication in respect of the findings resulting from the clinical study and its primary endpoint shall emanate from the coordinating investigator, principal investigators and other involved investigators in peer-reviewed journals and shall be presented at national and international meetings. The funding agencies (ZonMw and KCE) are also entitled to publish details of the selection process, the research objectives, plan and costs of the clinical study.

## Introduction

### Background and rationale {6a}

Traumatic brain injury (TBI) constitutes a major global health- and socioeconomic problem of insufficiently recognized proportions. Annually, 50 million TBI patients—including 2 million in Europe—are hospitalized, contributing to a total global burden of an estimated 400 billion US dollars [1, 2]. The incidence of TBI in elderly people (≥65 years old) has particularly increased over the past decades, especially in high-income countries, partly due to aging of the population [1]. This incidence is likely to increase even further because the share of elderly people in the EU is expected to double to 30% by 2060 [3]. In Belgium, an increased incidence of elderly TBI patients has already been reported, and in the Netherlands, the incidence of TBI was reported to be 213.6 per 1000 person years, highest in elderly [4, 5]. Improved healthcare has led to prolonged vitality of elderly people by decreasing morbidity from common diseases like cardiovascular diseases and cancer. However, this improved vitality also increases the risk of falling, which is the main cause of TBI in this age group [6, 7]. TBI in elderly patients also poses substantial economic challenges for the future, as the exponential increase in demand for medical care due to remodelling of the demographic pyramid is opposed by a reduction in available resources [1, 5].

The most frequently encountered pathological entity in TBI patients is an acute intracranial hematoma. Specifically, an acute subdural hematoma (ASDH) is the most common injury in elderly TBI patients and is often accompanied by a cerebral contusion [8, 9]. The enlarged subdural space of the progressively atrophic aging brain is associated with increased tension on bridging veins, making them more susceptible to shearing damage. Furthermore, the widespread use of anticoagulants and antiplatelet drugs among elderly increases the risk for ASDH development, even after low to moderate energetic head trauma [10]. Traditionally, older age has been closely associated to poor outcome after TBI [11, 12], partly because of comorbid illnesses negatively influencing outcome [12]. In reports from the 1980s and 1990s, mortality rates for patients aged ≥65 with an ASDH approached 90% [13, 14]. Hence, elderly patients have long been treated less “aggressively” compared to the younger patient population because of the presumed poor prognosis [10]. Over time, with faster transfers directly from accident to level I trauma hospitals, improved diagnostic tools and acute medical and intensive care, mortality rates in elderly patients with ASDH have declined from 90 to between 30 and 60% [15]. Along with this, neurologists’, trauma- and neurosurgeons’ traditional reserved attitude towards elderly patients has gradually shifted towards a more “aggressive” surgical approach [16, 17]. Despite this trend towards a more intensive treatment approach in the elderly sustaining TBI, the question whether to surgically or conservatively manage elderly patients with an ASDH remains a matter of huge controversy [18]. Surgical evacuation of the hematoma can be lifesaving, but is not necessarily restorative and may leave patients in a questionable quality of life, with huge costs to family and society [19–22]. Conversely, non-operative management may prompt favorable outcome in some patients, but can also result in—potentially preventable—death and disability [23, 24]. At present, it is not possible to accurately predict whether a specific patient will benefit from a certain treatment in terms of survival, functional recovery, and quality of life. This heterogeneity can be caused by many factors, including gender, anticoagulant/antiplatelet use, premorbid functioning, comorbidities, age, and the presence or absence of intracerebral contusions accompanying the ASDH. Current Brain Trauma Foundation (BTF) guidelines for the surgical management of TBI recommend the surgical removal of an ASDH measuring > 10 mm or when it causes a midline shift > 5 mm, regardless of a patient’s neurological state (Glasgow Coma Scale (GCS) score) [25]. These guidelines, released in 2006, are based on low-grade (class C) evidence from the 1990s and thus provide only limited guidance in modern clinical practice, allowing for subjective care and practice variation [23–26]. Indeed, great variation in BTF guideline adherence has been reported, ranging from 18 to 100% between studies [27, 28]. Moreover, major practice variation exists in Belgium and The Netherlands with regard to the management of traumatic ASDHs between centers, and even between neurosurgeons in the same center [29–31]. This variability in TBI management goes alongside unexplained variability in outcome and large between-center differences unrelated to case-mix [32–34].

For elderly patients, the uncertainties in clinical decision-making may be even worse, as they seem to represent a scientifically “forgotten group” in peer-reviewed literature. Patients aged ≥65 years have been excluded from most clinical studies on which current guidelines are based and thus no specific recommendations for this subgroup are stated [25, 35]. Likewise, the use of prognostic models in elderly is often limited due to lack of external validation in this group [19]. As a result, present day-to-day neurosurgical treatment for elderly patients with traumatic ASDHs is not based on high-quality evidence, but rather on a neurosurgeons’ training, experience, and clinical judgments, including a subjective estimation of the premorbid situation and vitality of the individual elderly patient. These judgments are made in the acute setting in considerable uncertainty, due to the absence of high-quality guidelines and accurate prognostication tools [36, 37]. This uncertainty covers the full spectrum of injury severity, as illustrated by two cases [29] (see below) of elderly patients with an ASDH, as examples out of a questionnaire sent to Dutch and Flemish neurosurgeons, in the context of mild and severe TBI (GCS > 12 and GCS < 9, respectively).

In *case 1*, 68.3% of neurosurgeons would evacuate the hematoma, while the rest would manage the patient conservatively. Those preferring surgery would presumably argue that acting in a too slow manner in case of a large ASDH would lead to neurological deterioration and death. On the other hand, neurosurgeons adopting a non-operative approach would not perform a risky operation without a more accurate estimation of the chance of neurological deterioration when surgery is withheld.

In *case 2*, 76.7% of neurosurgeons would surgically evacuate the hematoma, while 23.3% would choose a non-operative strategy. Those preferring surgery may hold the opinion that every patient deserves a chance, even if small, on survival and good recovery. Even though surgery is frequently not restorative in severely injured patients, recent studies have shown that the prognosis for elderly patients with severe TBI is not hopeless and that up to 40% of patients with an admission GCS of 3–4 survives and 11% even achieves favorable outcome after surgery [13, 38–41]. Nevertheless, those in favor of conservative management may hold the opinion that the outcome will be unfavorable, regardless of surgery. This thinking is in line with a recent article cynically titled “does a neurosurgeon rather fill nursing homes or cemeteries?” implying the choice to be between death and severe disability [42]. From this perspective, some physicians prefer comfortable end-of-life care in these patients, although this remains a reason for societal debate.

While opinions regarding the potential benefit of surgery may differ for case 2, it is generally agreed upon that non-operative management of severe TBI patients will inevitably lead to death (albeit with some exceptions described later), given the nature of the injury. This is illustrated by a recent study on ASDHs in octo- and nonagenarians in which all posttraumatic comatose patients treated conservatively died during hospital stay [13]. In conclusion, neurosurgeons must balance the risk of not doing enough against the risk of treating too aggressively, in both situations risking major consequences like death or poor neurological outcomes.

#### Existing research evidence

Of the limited postmillennial literature on (surgical) treatment of ASHDs in elderly, some have attempted to identify subgroups that may benefit from surgery [10, 17, 43–49]. However, these retrospective studies were performed in widely varying patient populations and may all suffer from (selection) biases due to their non-randomized nature. Those patients perceived as salvageable tended to be selected for surgery, while those considered “too sick to operate” tended to be treated conservatively, leading to self-fulfilling prophesies and skewing of the results [47]. Thus, no satisfactory conclusion regarding a preferred treatment strategy can be drawn from these studies. A recent study conducted by the authors, which compared treatment strategy on a center level rather than on a patient level to reduce confounding by indication, showed that an aggressive surgical management strategy was associated with better outcome in an elderly population with traumatic ASDHs [30]. Few studies have specified the type of intervention, and if they did, they did not address the effectiveness of the procedure [25]. Specifically, the decision to pre-emptively perform a decompressive craniectomy (DC) after evacuating the ASDH in an attempt to prevent increased intracranial pressure (ICP) due to brain swelling after surgery, is outweighed against the high morbidity and mortality of a DC, especially in the elderly population [50]. In the aforementioned questionnaire, as well as in a British survey, the choice for craniotomy (CR) or DC in patients with ASDH was shown to vary considerably [29, 51]. A randomized trial investigating DC versus CR for patients with traumatic ASDHs is currently in progress [52]. While this trial is likely to provide valuable information regarding the preferred operative strategy when surgery is considered indicated, it does not address the uncertainties in the decision-making process preceding the operation. Other large ongoing studies investigating the effectiveness of (surgical) treatment for TBI patients using “comparative effectiveness research” (CER) by evaluating observational cohorts are the international CENTER-TBI and the Dutch Net-QuRe initiatives. CER has been considered an elegant method to circumvent the difficulties of performing randomized clinical trials by making use of the existing local practice variation. However, increasing skeptics argue that CER does not take individual patient differences into account and may result in over-rationing of healthcare driven by financial considerations [53]. Being scientific participants in both CENTER-TBI and Net-QuRe, the investigators observe that the great uncertainty regarding the optimal treatment of traumatic ASDHs in elderly patients will not be solved by the currently ongoing CER studies. In line with this, a recent study on surgical versus conservative management of ASDHs emphasized the need for more data from larger populations to obtain definite results [54].

#### Proposed study rationale

The RESET-ASDH investigators propose a randomized controlled trial on the role of early neurosurgical hematoma evacuation versus non-operative management in elderly patients with a traumatic ASDH. The research group acknowledges that there is a particular subgroup of patients presenting with very poor clinical and radiological parameters, in which randomization of treatment may not be ethically justifiable. The fact that the ethical desirability of surgery in these severely injured patients may be questionable given the high chance of death or unfavorable outcome (which is the reason for some neurosurgeons to opt for conservative management of the aforementioned patient in case 2), does not change that conservative management of these patients equals imminent death. Questioning the effectiveness of surgery in these patients has been compared to questioning the effectiveness of a parachute in jumping out of a plane [55, 56]. Therefore, the most severely injured (defined as a GCS < 9) patients are, in the opinion of the researchers, unsuited for randomization and will not be included in this study with the exception of a specific subgroup described next. There is a category of comatose ASDH patients with a relatively small hematoma less than 10 mm thick and with less than 5 mm midline brainshift and no extracranial explanations for their coma (e.g., internal or external hemorrhages resulting in hypovolemia, hormonal or electrolyte imbalances, infections, and toxic substances) as assessed by the treating staff neurosurgeon. The most likely explanation is diffuse brain injury, not (yet) apparent on a CT scan. The surgical challenge lies in the decision to evacuate the hematoma in an emergency operation with the possibility that the hematoma is not the main explanation for the increased ICP or comatose condition. The BTF guidelines recognize this dilemma and point out that a prospective study should compare an aggressive strategy versus non-operative management (with the possibility of secondary surgery after neurological deterioration) in these comatose patients. Hence, an important subgroup of comatose patients with a small traumatic ASDH < 10 mm and a midline shift < 5 mm will be included in the randomized study.

Thus, those patients for whom scientific controversy exists regarding the acute management and randomization is ethically acceptable are potential candidates for this study. However, even among these patients, there may be individual cases in which the neurosurgeon strongly prefers a certain treatment. As this study is pragmatic, it is not feasible to randomize such patients against the treating neurosurgeon’s best intention. Therefore, only patients for whom the treating neurosurgeon is in *equipoise* about the benefits of early surgery compared to initial non-operative treatment will be eligible for this trial. A recent trial on traumatic intracerebral hematomas (STITCH) used a similar “clinical equipoise” design, but was prematurely halted by the funding agencies due to concerns about insufficient patient recruitment in the UK [57]. The majority of centers participating in the RESET-ASDH study have proven to adequately include patients in large prospective projects concerning surgical versus conservative treatment strategies (CENTER-TBI, Net-QuRe, Sciatica Trial, Sciatica MTD, Sciatica PLDD and the current PTED study) and are prepared to continue to do so in the future. Furthermore, the inclusion and consent procedures as described in this proposal are expected to result in an effective recruitment of sufficient patients.

In conclusion, the role of operative versus conservative management in elderly patients with traumatic ASDHs and the associated long-term functional outcomes and costs remain largely elusive. Hence, the RESET-ASDH researchers propose a prospective randomized controlled trial in elderly patients with a traumatic ASDH for whom scientific controversy and clinical equipoise exists regarding the preferred treatment strategy. The following research questions will be answered:

1. Is early neurosurgical hematoma evacuation in elderly patients with a traumatic ASDH more (cost-)effective than a conservative (wait-and-see) management?
2. Is it possible to identify subgroups of patients who will benefit substantially in interaction with one of the proposed treatment strategies? (e.g., pre-trauma use of anticoagulants or not, surgery with bony decompression versus without decompression, ICP monitoring or not)

The authors hypothesize that early neurosurgical hematoma evacuation generally leads to a better functional outcome (GOS-E) and is more cost-effective compared to conservative management, although subgroups may be identified for which the latter is the preferred treatment strategy.

#### Possible return of investment (ROI)

The financial consequences of TBI for individuals and society are substantial in both Belgium and The Netherlands. TBI is associated with significant direct healthcare costs in terms of pre-hospital care, emergency care, hospitalization, long-term post-discharge care, and rehabilitation, as well as indirect costs, i.e., due to loss of productivity of both patient and family [5]. The total direct and indirect costs of TBI in Europe were estimated to €33 billion [58]. In 2012, estimated annual costs of TBI in the Netherlands were €314.7 million, with €158.8 million direct and €155.9 million indirect costs [5]. The mean total costs per TBI case were €18,030 [5]. Since then, these costs have probably increased. Recent work from the investigators shows that mean in-hospital costs were €24,980 per ASDH patient and primarily the result of costs related to admission (€14,980) and surgical intervention (€6,890) [59]. In this regard, a large amount of costs could be saved if early surgery turns out not to be more effective than a conservative treatment strategy. On the other hand, if surgery turns out to be more effective, an incremental cost-utility analysis could prove surgical treatment to be the most cost-effective strategy. In line with this, a study has shown that aggressive surgical management of severe TBI in patients aged 60–80, despite being the most expensive treatment strategy, was also the most cost-effective strategy on the long term because it resulted in better outcome and thus lower costs associated with long-term nursing care and lost productivity [60, 61]. In fact, this is probably an underestimation of the true effect because current methods of cost analysis are not sensitive enough to capture the contributions (for example, familial childcare or retirement spending habits) of the older population to society. Similarly, the loss of productivity of caregiving family members is probably overlooked in most analyses. The investigators propose to assess the cost-effectiveness of treatment by performing a cost-effectiveness analysis (CEA) and a cost-utility analysis (CUA). The results of these health-economic analyses will lead to more cost-effective treatment of this rapidly increasing patient group in an economically challenged healthcare system. They will give insight in the magnitude of the problem and quantify the cost-effectiveness of complex trauma care in the fragile elderly, which have been excluded in previous studies. This study holds great potential for return of investment, especially since the implementation percentage is expected to be high because high-quality evidence is lacking and clearly awaited. In any case, the results of this trial will better inform societal health-economic discussions and improve health-economic moral deliberations.

### Objectives {7}

#### Primary objective

The primary objective is to establish the effect of early surgical hematoma evacuation compared to conservative treatment on functional outcome (as expressed by the GOS-E) after 1 year in elderly patients with a traumatic ASDH (Table 1).

**Table 1:** The GOS-E score

GOS-E	Category	Description
1	Dead	
2	Vegetative	Condition of unawareness with only reflex responses but with periods of spontaneous eye opening
3	Lower severe disability	Patient fully dependent for all activities of daily living. Requires assistance to be available constantly. Unable to be left alone at night
4	Upper severe disability	Can be left alone at home for up to eight hours but remains dependent. Unable to use public transport or shop by themselves
5	Lower moderate disability	Able to return to work in sheltered workshop or non-competitive job. Rarely participates in social and leisure activities. Ongoing daily psychological problems (quick temper, anxiety, mood swings, depression)
6	Upper moderate disability	Able to return to work but at reduced capacity. Participates in social and leisure activities less than half as often. Weekly psychological problems
7	Lower good recovery	Return to work. Participates in social and leisure activities a little less and has occasional psychological problems
8	Upper good recovery	Full recovery with no current problems relating to the injury

#### Secondary objectives

- Functional outcome as expressed on the GOS-E besides the 1 year measurement (this includes mortality)
- Disease-specific quality of life as expressed on the QOLIBRI
- Health-related quality of life as expressed on the EuroQol-5D-5L
- Cognitive functioning as expressed on the MOCA
- Direct and indirect costs
- Duration of hospital stay
- Time from event to surgery
- Discharge locations
- Complications (during hospital stay)
- Secondary surgery in both groups

### Trial design {8}

The RESET-ASDH trial is a pragmatic, multicenter, randomized controlled trial, comparing 2 different treatment strategies in elderly patients with a traumatic ASDH: early surgical hematoma evacuation versus conservative treatment. The study is designed to evaluate the superiority of surgery on functional outcome (GOS-E) at 1 year compared to conservative treatment; hence, a superiority trial design was applied. Patients will be recruited for the study and randomized to one of the treatment arms if scientific controversy exists regarding the acute management, randomization is ethically justifiable, clinical equipoise is present, and informed consent is obtained or deferred. The study will include a 1-year follow-up period during which outcomes will be assessed at 3 months, 6 months, and 1 year by a visiting member of the research team (research nurse) and a long-term follow-up with annual questionnaires digitally, by telephone or postal for up to 5 years after the initial trauma. Patient inclusion is expected to be completed in 2 years. The estimated duration of the study (without long-term follow-up) will be 3 years.

#### Degree of pragmatism

**Fig. 1:**
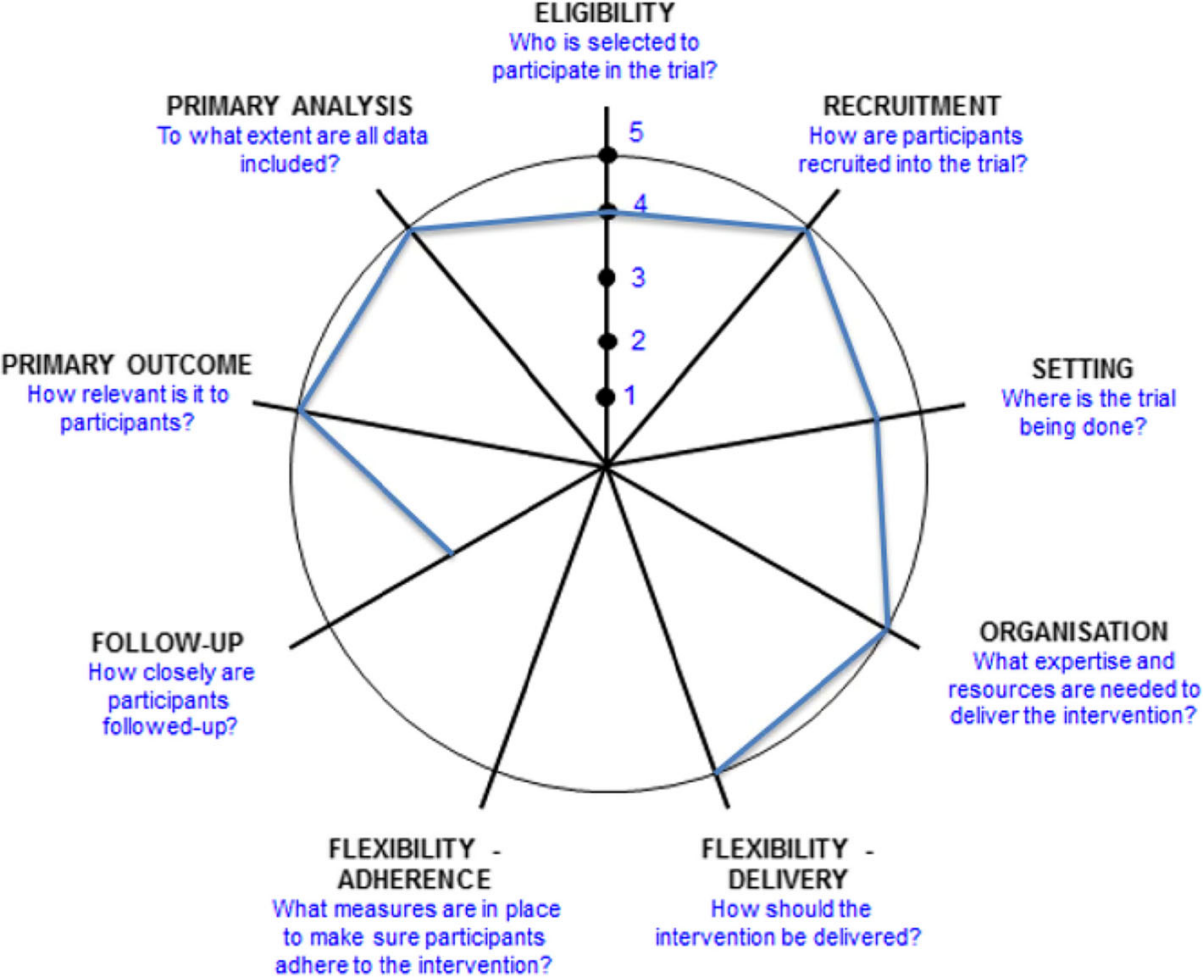
PRECIS-2 wheel for RESET-ASDH trial

The RESET-ASDH study is designed to gather real-world evidence that is applicable to routine clinical practice in Belgium and The Netherlands. The degree of pragmatism in this study was assessed by the PRECIS-2 tool (Fig. 1, Table 2) [62].

**Table 2:** PRECIS-2 scores for trial domains

Domain	Score	Rationale
Eligibility criteria	4	The participants in the trial accurately represent the patients who would receive one of the treatments in usual care, as it concerns all elderly with a traumatic ASDH for whom clinical equipoise exists except for the most severely injured patients, as these are not suited for randomization due to ethical reasons.
Recruitment path	5	There will be no overt recruitment effort as patients will be recruited in the usual clinical care setting.
Setting	4	The trial will be conducted in the setting of daily clinical practice in multiple academic and peripheral hospitals in Belgium and The Netherlands spanning a large geographical area, leading to a realistic cross-sectional patient population of both countries. However, the large variability in neurotrauma care between countries, even within Europe, partly hampers generalizability beyond Belgium and The Netherlands.
Organization intervention	5	The trial will compare two treatment modalities that are already widely applied in current clinical practice as standard treatments in all participating hospitals. Therefore, only existing diagnostic procedures, healthcare staff and resources are necessary for the interventions under investigation.
Flexibility of intervention - delivery	5	The details of the treatments under investigation, including the specifics of the surgical procedure as well as the conservative-medical management protocol will be left up to the participating centers. Thus, the trial takes the existing variability in usual care between centers into account and therefore allows for flexibility in delivery of the intervention and implementation of the results.
Flexibility of intervention - adherence	Not applicable	As this is a surgical trial, there is no adherence issue after patients are randomized to either surgical intervention or initial conservative treatment with the possibility of delayed surgery in case of neurological deterioration. Therefore, this domain of the PRECIS-2 is not applicable in this trial.
Follow-up	3	Follow-up visits are more frequent and more intense (i.e., more data is collected per follow-up visit) than would be typical under usual care. However, follow-up in this surgical trial will not result in care management that differs from usual care (i.e., it is not possible for follow-up visits to have an impact on treatment engagement and it is highly unlikely that they would effect response to treatment). Therefore, the longer and more intense follow-up in this trial is not inconsistent with a pragmatic approach.
Outcome	5	The primary outcome measure is highly relevant from a patient’s perspective, as it scores functional outcome including mortality. Moreover, the primary outcome of this trial (as well as secondary outcomes) was chosen after extensive discussions between the investigators and representatives of relevant patient organizations, patients and their caregivers. Secondary outcomes including the economic analyses will also be meaningful to policymakers in both Belgium and The Netherlands.
Analysis	5	All data will be analyzed according to an intention-to-treat analysis. Also, a proportional odds regression model will be used, which is a more sensitive method compared to traditional dichotomized analyses and allows more data to contribute to the analyses.

This trial can be justly labeled as pragmatic as it scores a 4 or 5 on most domains of the PRECIS-2 tool and does not score lower than a 3 on any applicable domain [63].

## Methods: participants, interventions, and outcomes

### Study setting {9}

Elderly patients (≥ 65 years old) presenting to participating Dutch and Belgian hospitals during the 2-year inclusion period of this trial are potentially eligible for this trial. The participating centers form a balanced representation of 8 Belgian and 8 Dutch centers and within Belgium both Dutch- and French-speaking centers are represented. All participating hospitals are experienced clinical trial centers and will include a wide variety of patients with different backgrounds and cultures. With large level-1 university trauma centers in Brussels, Antwerp, Rotterdam, The Hague, and Nijmegen, the research group is acting in a multi-cultural society, besides the old University regions of Leuven and Leiden. This will lead to a realistic cross-sectional patient population of Belgium and The Netherlands, which makes the later study results as well as possible change in guidelines generalizable to other populations and easier to implement. An up-to-date list of all study sites can be obtained from the “Nederlands Trial Register (NTR)” or “ClinicalTrials.gov” (https://clinicaltrials.gov/ct2/show/NCT04648436?term=NCT+04648436&draw=2&rank=1).

### Eligibility criteria {10}

#### Inclusion criteria

To be included in the RESET-ASDH trial, the following conditions must be met:

- Age ≥ 65 years
- A GCS of ≥9 and a traumatic ASDH > 10 mm in diameter or a traumatic ASDH < 10 mm but with a midline shift* > 5 mm, or a GCS < 9 and a traumatic ASDH < 10 mm and a midline shift* < 5 mm without extracranial explanations for the comatose condition
- Clinical equipoise exists (i.e., the responsible neurosurgeon admits there is insufficient certainty based on evidence about the benefits of either treatment)
- Informed consent is obtained or deferred (see item 26a)

* Midline shift will be measured as the perpendicular distance between the septum pellucidum and a line designated the midline on CT scan in brain setting.

**Fig. 2:**
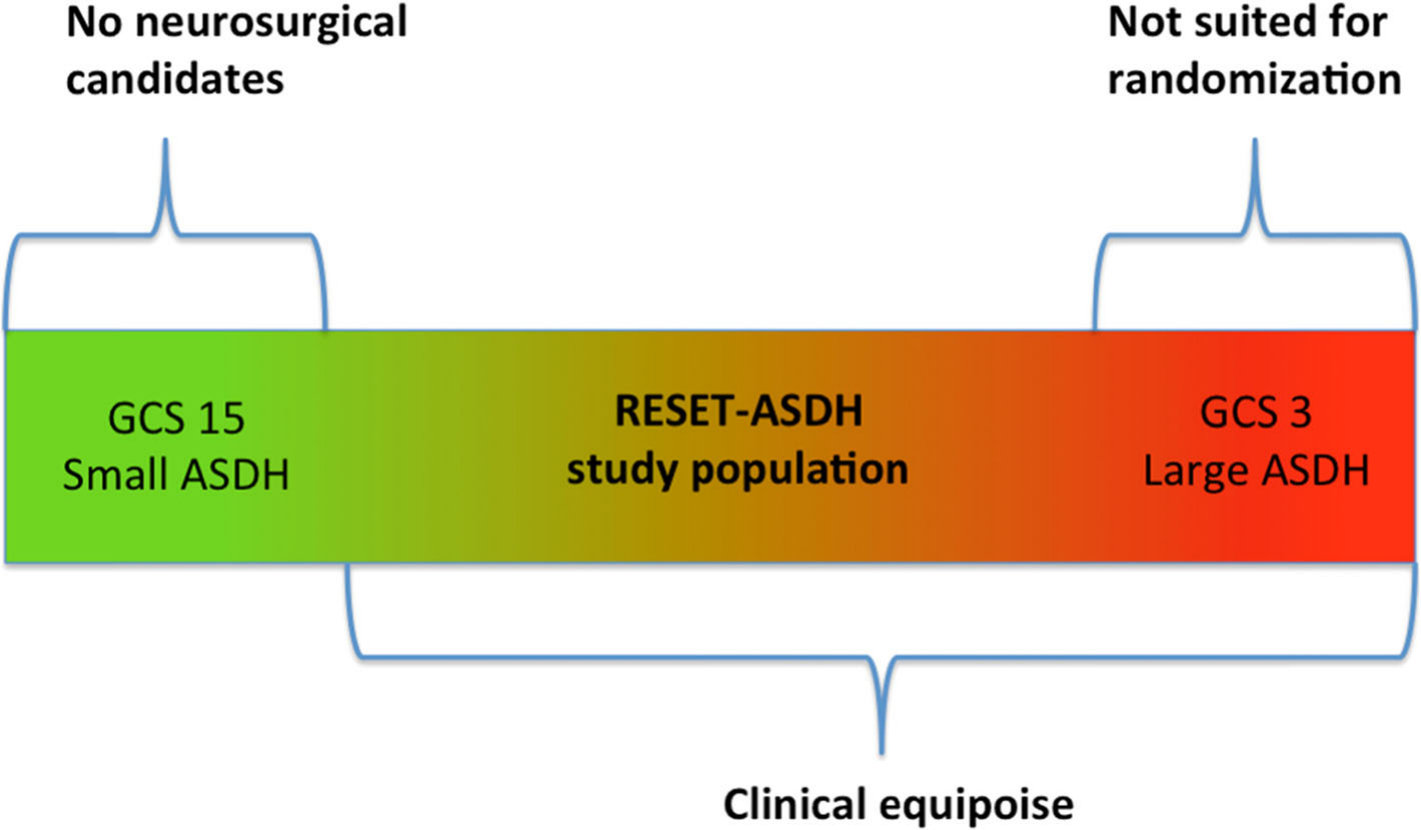
RESET-ASDH study population

Importantly, due the complexity and heterogeneity of the injury under investigation, the target population for the RESET-ASDH study cannot be conclusively defined by rigid criteria. The abovementioned GCS scores and hematoma sizes are meant to provide a framework based on scientific equipoise and existing guidelines within which the treating neurosurgeon can decide on clinical equipoise, which is of major importance in this pragmatic trial. Figure 2 displays the gradual nature of injury severity and illustrates the target population for the RESET-ASDH study.

Clinical equipoise, caused by scientific uncertainty and lack of evidence, can be a difficult subject for surgically trained MDs as they are educated to not let uncertainty influence their acute decision-making. Due to international epidemiological training of neurosurgeons, they are becoming more familiar with clinical equipoise, as well as with the window within which randomization between different treatment strategies—including surgery—can occur. All neurosurgical-, trauma- and neuro-ICU staff participating in the RESET-ASDH study and involved in the acute care of neurotrauma patients will be trained by the sponsor on location prior to trial start by means of case-based tutoring sessions. The Dutch PI has ample experience with this as it was done similarly prior to other large randomized studies comparing a surgical strategy with initial conservative treatment [64, 65].

#### Exclusion criteria

A potential subject who meets any of the following criteria will be excluded from participation in this study:

- Additional epidural hematoma (EDH) or infratentorial (e.g., cerebellar) intracerebral hemorrhage (ICH)
- Major traumatic abdominal or thoracic injury (each separately defined as an Abbreviated Injury Scale (AIS) score ≥ 4) [66, 67] or a “moribund” state at presentation (e.g., bilaterally absent pupillary responses)
- Known terminal condition resulting in a life expectancy of less than 1 year
- Severe and progressive dementia or cerebral infarction necessitating daily care in a nursery home in the pre-trauma period

### Who will take informed consent? {26a}

#### Recruitment and consent

Patients will be recruited from the emergency departments of multiple large hospitals in the Netherlands and Belgium. Potentially eligible patients will ideally be asked to provide written informed consent by their treating physician. If they are not capable to do so, a legal representative will be asked to provide surrogate consent for the patient. Unfortunately, surrogates are mostly unavailable in the acute moment [68]. Also, the time critical nature of starting acute treatment does often not allow for extensive consent discussions with legal representatives, even if they are present. For the conservative treatment group, rapidly starting medical therapy to reduce ICP, if necessary, is considered important [69]. Similarly, surgical treatment is considered to be most effective when performed as soon as possible [25]. If there is insufficient time to discuss consent with a legal representative prior to starting necessary treatment, the treating clinician will take responsibility for including the patient using “deferred consent” [70] provided the necessary conditions (listed below) are met and consent type is documented in the electronic patient file. The justification for the deferred consent procedure is the clinical equipoise of both interventions and therefore the absence of extra risk for the patient, but also the emergency of the intervention and the group relatedness (meaning that most of the benefits from the study are applicable to future patients with a similar condition).

**Fig. 3:**
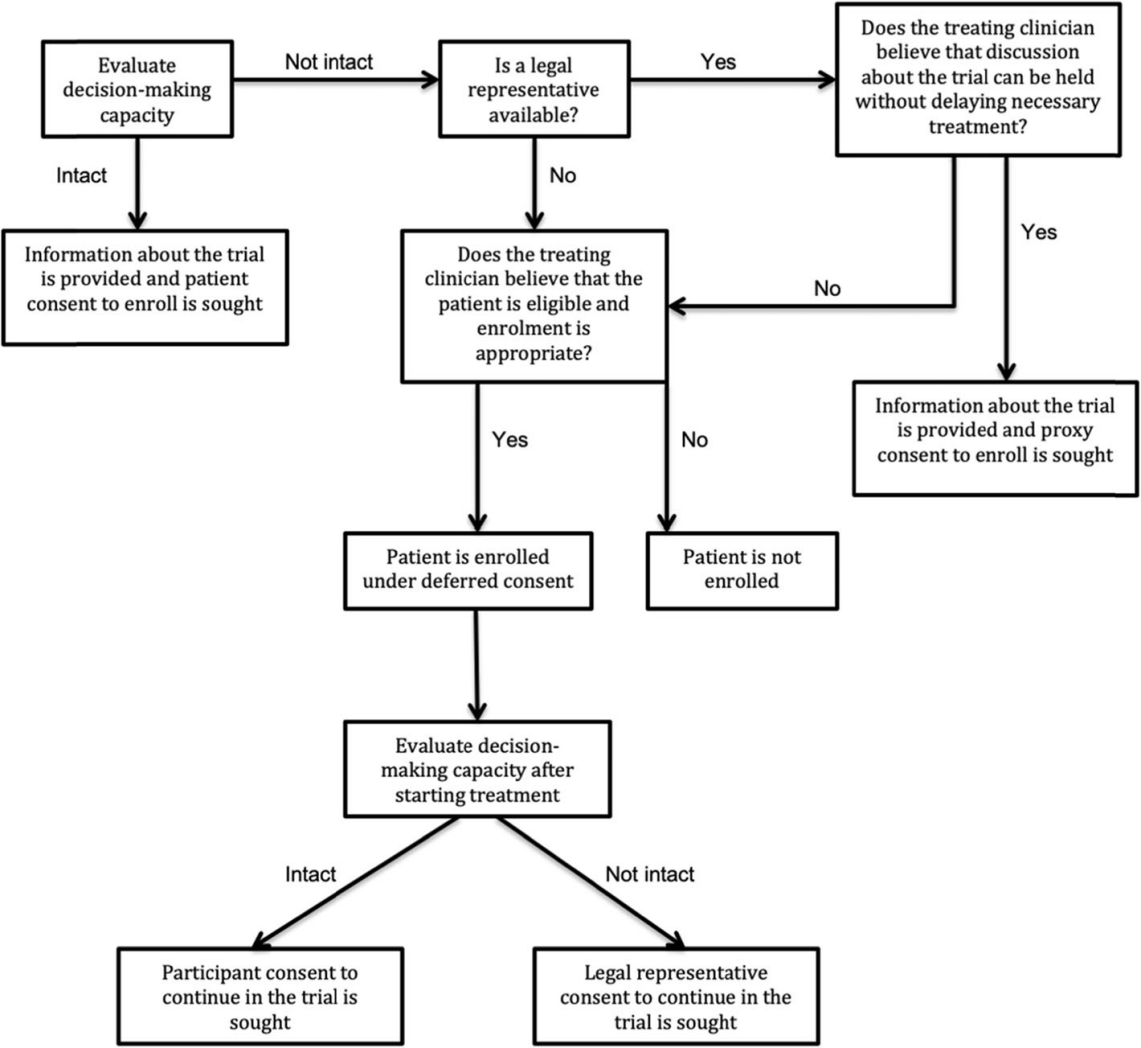
Consent algorithm RESET-ASDH trial

The following procedures, also shown in Fig. 3, will be followed for obtaining consent and enrolling patients into this trial.

#### Enrolment in the trial with patient consent

Ideally, the treating physician will go through a written information sheet together with the patient and will allow as much time as possible to discuss the treatment options and the aim of the study, while at the same time making sure that medical treatment is not unnecessarily delayed. Every step will be taken to ensure that a test of capacity is undertaken before a decision on a person’s capacity to consent or not to consent to participation in research is taken. When the patient is considered capable to consent and the consent form is signed, one copy will be given to the patient, one will be filed in the patient notes, and one will be filed in the trial documentation. The patient is subsequently enrolled in the trial. If the patient refuses participation, his or her wishes will be respected.

#### Enrolment in the trial with proxy consent

In case the patient is unable to give consent him- or herself due to the nature of the injury (i.e., altered consciousness), all efforts will be made to locate a legally acceptable representative to serve as a surrogate decision maker (e.g., consultee, proxy, next of kin) on behalf of the patient. The surrogate decision maker can be a mentor or someone with a close personal relationship with the patient who is capable and willing to consent on behalf of the patient according to Dutch Civil Law (Burgerlijk Wetboek artikel 7:465). If the patient has been placed under guardianship or mentorship, surrogate consent should be given by the curator or mentor. In other cases, surrogate consent should be provided by a person who is authorized in writing by the patient to act in his/her place. If such a person is missing or if this does not occur, surrogate consent should be provided by the patient’s spouse or other life companion unless this person does not wish this, or, if such a person is also missing, by a parent, child, brother, or sister, unless this person does not want this. If time allows and a legal representative is available, the treating clinician will have a discussion explaining the nature of the condition, the treatment options, and the aim of the study. This discussion may take place face-to-face but may also take place over the telephone as neurosurgical units cover relatively large geographical areas and there is not always enough time for a legal representative to get there in time. Both the treatment provided and the patient’s participation in the study, which are sometimes but not necessarily linked, will be discussed. If present, the legal representative will be asked to sign the proxy declaration form. If the legal representative objects to the inclusion of the patient in the trial, his/her views will be respected.

#### Enrolment in the trial with deferred consent

Unfortunately, as TBI often occurs outside the domestic situation, family members are rarely available during the first hours after trauma [68]. In cases where a legal representative cannot be traced or there is no time to discuss trial participation with them prior to starting treatment, the treating clinician will take responsibility for entering the patient into the trial under “deferred consent” provided the following conditions are met:

- The patient is in a potentially life-threatening situation, treatment is required without delay
- Urgent treatment is not possible to separate from inclusion in the trial
- The two procedures under comparison in this trial (early surgery and conservative management) are both well established, routine procedures in the treating center
- The risks for the patient involved in participating are considered no larger than the risks involved in not participating
- The patient meets the eligibility criteria for trial entry

Depending on the decisional capacity of the patient after starting treatment, every effort will be made to inform the patient and ask for consent to continue trial participation or trace/contact a legal representative and provide him/her with information on the trial and seek his/her agreement to continue participation in the trial. If the patient or the legal representative refuses for whatever reason, the participant will be withdrawn and no further data will be collected.

#### Participants regaining capacity

If participants regain capacity while in the hospital, they will be given information about the clinical trial and their consent will be sought to continue in the trial. If the patient withdraws his or her permission (informed consent), the patient data will not be used for this study.

If the patient does not recover enough to provide his or her own informed consent, then the deferred consent or the consent of the legal representative will continue to apply. The research team will reassess the patient’s decisional capacity during all subsequent follow-up visits.

#### Waiver of consent

In case the patient dies before regaining capacity and before a legal representative can be contacted, retrospective consent from the legal representative for trial entry will not be sought and the patient will be included under “waiver of consent” according to Dutch Civil Law (Burgerlijk Wetboek artikel 7:458 lid 1 en lid 2), unless the patient has objected against the use of his/her data (per 7:458 lid 2 sub c). In Belgium, consent will be obtained from a legal representative, since “waiver of consent” is not supported by Belgian law. In The Netherlands, legal representatives have no independent right on inspection of or say on therapeutic or study data (CCMO: De nabestaanden hebben geen zelfstandig recht op inzage van de tijdens de behandeling en het onderzoek verkregen gegevens en hebben daar ook geen zeggenschap over. Van toestemming voor het gebruik van de data door de nabestaanden kan daarom ook geen sprake zijn) [71]. Also, possible refusal by the legal representative may cause selection bias which is ethically unwanted (CCMO: het introduceren van selectiebias door het moeten vragen van toestemming aan de nabestaanden, mocht daar grond voor zijn, ethisch niet wenselijk is) [71]. Use of the data has no implications for the patient or legal representatives. Furthermore, the investigators find it unethical to burden the grieving relatives with a decision that has no impact on the already performed treatment and only pertains the use of already gathered coded data. The legal representatives will be given a letter containing information about the trial. They will also receive an invitation for an appointment with the supervising doctor and an investigator after 6 to 8 weeks to answer any remaining questions.

#### Ethical justification

The investigators believe that the suggested approach meets the requirements of the Declaration of Helsinki, as it will ensure that:

- Despite possible lacking capacity to consent and time pressure due to the injury, elderly patients with traumatic ASDHs can still be enrolled in a trial that aims to answer an important question that will advance the treatment of future patients.
- If the patient is capable, a discussion about the trial will be had before start of treatment.
- If the patient is not capable and a legal representative is available, a discussion about the trial will be had before start of treatment as long as the treating clinician believes this would not delay treatment.
- If the patient is not capable and the treating clinician believes there is not sufficient time to discuss the trial with a legal representative prior to starting treatment, enrolment of the patient will be possible under “deferred consent” as long as the necessary conditions listed in this protocol are met and documented.

#### Objection by minors or incapacitated subjects

If subjects are (temporarily) incapacitated, they can be included in the trial by deferred/surrogate consent depending on the presence of legal representatives and time pressure to start treatment, according to the conditions listed in this protocol. Every effort will be made to reassess the patient’s decision-making capacity and/or to trace a legal representative after starting treatment and provide him/her with information on the trial and seek his/her consent to continue participation.

#### Benefits and risks assessment, group relatedness

##### Benefits

Participants will receive more detailed and more intensive follow-up visits after treatment. They will be informed about the results of the research as soon as they are available, and these results will also be shared with the clinical team responsible for their medical care. However, participating in this study does not mean a patient’s outcome will be better when compared to not participating. The benefits are expected to be mostly for future patients. In conclusion, by participating, patients are contributing to better medical care for future elderly patients with a traumatic ASDH, as well as to a broader knowledge base about this rapidly increasing medical and economic problem.

##### Risks

Two treatment modalities that are already applied in current clinical practice as standard treatments (early surgery versus initial conservative treatment) are randomized in this study. The risks are therefore expected to be no higher for patients participating in the study than for patients outside of the study. Study participation adds a minimal burden of three follow-up evaluations in the first year (at 3, 6, and 12 months) and subsequent yearly evaluations by phone or postal until 5 years after the injury.

### Additional consent provisions for collection and use of participant data and biological specimens {26b}

This is not applicable; no biological specimens are collected.

## Interventions

### Explanation for the choice of comparators {6b}

The surgical versus initially conservative treatment of elderly patients (≥ 65 years old) with a traumatic ASDH remains an important clinical and moral dilemma. Current BTF guidelines are not based on high-quality evidence and compliance is low, allowing for large (inter)national treatment variation. The RESET-ASDH trial is an international multicenter RCT on the (cost)effectiveness of early neurosurgical hematoma evacuation versus initial conservative treatment in elderly with a t-ASDH. The principle of randomizing neurosurgical patients to conservative treatment versus surgery has already been proven by the earlier neurotrauma studies DECRA trial [72], the STITCH trial [73], and the RESCUEicp trial [50]. Furthermore the current researchers have experience with this design in randomized surgical studies like The Sciatica Trial and the DECSA trial [64, 65].

### Intervention description {11a}

#### Study procedures

##### Early surgical hematoma evacuation (group A)

Patients randomized to group A will undergo rapid (preferably within 1 to 2 h after randomization with a maximum of 8 h) neurosurgical evacuation of the ASDH with or without decompressive craniectomy (DC) (i.e., leaving out the bone flap). Generally in Europe, a craniotomy is performed for hematoma evacuation and DC when (intractable) swelling is seen intra-operatively or when swelling is expected (preventive). The general techniques are described below:

*“After induction of general endotracheal anesthesia the patient is positioned on the back with the head placed in a lateral position with the unaffected side towards the ground. The head is secured in a three-pin Mayfield skull clamp. After the scalp is appropriately sterilized and draped, a curvilinear incision is made through all tissue layers to allow exposure of the appropriate entry on the skull bone. The skin- and muscle flap are lifted off the bone and folded frontobasally. Burrholes are made with the electrical drill and a boneflap is formed with the craniotome or a Gigli-saw by connecting the burrholes. The dura is cut along the bone edges. Next, the ASDH presents itself and is removed from beneath the dura in all corners. The removal of the hematoma may be facilitated by irrigation with water. After removal of the hematoma to the satisfaction of the surgeon’s discretion, hemostatic measures are taken with coagulation of potentially present bleeding cortical veins. Retractors are removed and the dura is closed with sutures. In case no DC is performed, the bone flap is replaced back in its original confines and secured to the skull with titanium plates and screws or with sutures. In some cases, a drain is placed under the skin for drainage of blood or fluid from the surgical area. The muscles and skin are sutured back together. A turban-like or soft adhesive dressing is applied. Generally magnifying loupes are used on the discretion of the surgeon. In the event of DC, the bone defect is generally made larger and the resulting bone flap will not be replaced. A bone flap of at least 11 cm anteroposterior (AP) diameter is raised. The decision for a DC can be made primarily or secondarily by increasing the defect of the bone flap that is formed during a normal craniotomy. In most cases this will be a unilateral frontotemporal, a parietal or a wide frontotemporoparietal craniectomy. Further surgical options are the replacement of (a portion of) the dura with autologous fascia, homologous tissue or synthetic material, and applied as a sutured graft or as an onlay. These nearly similar techniques are left to decide on the discretion of the surgeon. In conjunction to these surgical procedures the neurosurgeon can decide to place an intracranial pressure (ICP) monitor. The ICP device can be an intraparenchymal sensor or an extraventricular drain with a transducer for the ICP. The latter has an option to drain CSF (and thereby lower ICP). On a standardized form will be noted which technique is used and what the main findings were during surgery.”*

The operation will be performed by a qualified neurosurgeon or a sufficiently trained senior resident-in-training under supervision of staff. The general postoperative care on the ward or intensive care unit does not differ between Belgian and Dutch centers.

1. Craniotomy (CR) with or without dural grafting*
  This includes replacement of the boneflap.
2. Subtemporal decompressive craniectomy (DC) with or without dural grafting
  This includes removing part of the skull beneath the temporal muscle.
3. Large fronto- or temporoparietal decompressive craniectomy (DC) with or without dural grafting
  This includes removing a larger part of the skull from different areas.

(I) ICP monitoring and cerebral perfusion pressure (CPP)-guided ICU treatment
(II) No additional ICP monitoring

This includes placement of an intraparenchymal (within the brain parenchyma) sensor or extraventricular drain** with ICP transducer

* Dural grafting: the placement of a synthetic or biological graft to ensure dural closure

** A small tube surgically inserted into the brain ventricles, which can drain cerebrospinal fluid (CSF)

As this trial is pragmatic, the exact neurosurgical technique will be left to the discretion of the surgeons and local stand protocol and no efforts will be made to standardize these methods. However, the effect of the different surgical techniques on outcome will be analyzed.

##### Initial conservative management (group B)

Patients randomized to group B will be conservatively managed on a clinical medium neurocare ward or intensive care unit using a TBI treatment protocol based on the BTF. On the ICU, the diagnostic and therapeutic options include ICP monitoring with medical management of intracranial hypertension (i.e., hyperosmolar therapies, hyperventilation) and CPP guided treatment. On the ward, monitoring increased ICP by clinical observation can include waking the patient on predefined time points (every hour during the first 24 to 48 h). Patients initially randomized to group B, who experience significant neurological deterioration, defined as a decrease of GCS score of ≥3 points, will receive delayed secondary hematoma evacuation after a repeated CT scan as deemed necessary by the treating neurosurgeon and will stay within group B as this is an ITT design. Similarly, group B patients who undergo secondary burr hole drainage within 6 months after initial trauma will be analyzed as a subgroup within group B. Alternatively, on the discretion of the treatment team (neurosurgeon, ICU doctor, and neurologist) and communication with the family it can be decided that “comfortable end-of-life care” is more appropriate than surgery. The principle that “the interest of the patient always prevails over those of science and society” will apply in this study.

##### Observational cohort (group C)

An observational cohort group (C) containing all elderly patients with a traumatic ASDH presenting to one of the participating centers during the inclusion period, including those who meet the exclusion criteria for randomization or die before randomization, will be registered parallel to the randomized groups in the form of a screening log.

### Criteria for discontinuing or modifying allocated interventions {11b}

Subjects can leave the study at any time for any reason if they wish to do so without any consequences. In case of withdrawal at any point during the hospital phase, treatment will be provided according to standard local clinical practice. The investigator can decide to discontinue the treatment to which a subject was randomized for urgent medical reasons.

As described in item 11a, patients initially randomized to group B can receive delayed secondary hematoma evacuation within the study protocol in case of neurological deterioration.

### Strategies to improve adherence to interventions {11c}

This is not applicable; as this is a surgical trial, there is no adherence issue after patients are randomized to either surgical intervention or initial conservative treatment with the possibility of delayed surgery in case of neurological deterioration. As described in items 11a and 11b, cross-over of patients from group B to group A is possible within the study protocol.

### Relevant concomitant care permitted or prohibited during the trial {11d}

The details of the treatments under investigation, including the specifics of the surgical procedure, the conservative-medical management protocol and concomitant care will be left up to the participating centers. Thus, this pragmatic trial takes existing variability into account and therefore allows for flexibility in delivery of the intervention and implementation of the results. Importantly, the general postoperative care on the ward or intensive care unit is not expected to differ substantially between Belgian and Dutch centers.

### Provisions for post-trial care {30}

The local site retains all responsibility, medical and otherwise, to provide the best care for their patients. The sponsor has a liability insurance (Centramed, Maria Montessorilaan 9, 2719 DB Zoetermeer, The Netherlands) which is in accordance with article 7 of the WMO. The Medical Ethical committee Leiden-The Hague has waived the obligation to take out an additional study subject insurance due to the absence of additional risk involved in participation.

### Outcomes {12}

#### Main study parameter/endpoint

The primary endpoint will be the Extended Glasgow Outcome Scale (GOS-E) at 1 year after injury [74]. The use of the GOS-E as a core global outcome measure is recommended by the interagency TBI Outcomes Workgroup and the International Mission for Prognosis and Analysis of Clinical Trials in TBI group (IMPACT Common Data Elements) [75]. The GOS-E [76], derived from its precursor the GOS [77], is globally the most commonly used TBI outcome measure. While the GOS grades disability on a 5-point scale and is determined largely by physical deficits, the GOS-E provides a higher sensitivity by defining disability on an 8-point scale and incorporating emotional and cognitive disturbances affecting disability. Especially in the elderly emotional and cognitive disturbances are described after undergoing complex cranial surgery, in particular in ASDH.

The GOS-E is designed as a structured interview and can also be applied through telephone [78] and e-mail [79]. This allows for long-term follow-up without a high burden for patients. Although several other primary outcome measures for TBI exist, the GOS(-E) remains the most widely implemented and best validated tool to assess outcome in TBI and permits comparison to much of the world literature on TBI outcome [80, 81]. In the (retired) elderly population, the GOS-E should be interpreted with reference to previous engagement (including reintegrating to former social and leisure activities instead of work per se) and the extent of any change, as in most people in this aged population there is absence of paid employment. Experienced research nurses will grade outcomes based on the GOS-E in each patient according to a standardized approach [76].

#### Secondary study parameters/endpoints

Secondary outcomes will be measured at 3, 6, 12, 24, 36, 48, and 60 months after randomization. Follow-up at 3, 6, and 12 months will be executed by live visits from research nurses from Leiden/Leuven. Other follow-up moments will be captured by postal or telephone, depending on the clinical state of the patient. As the GOS-E is a global outcome measure, cognitive, physical, social, and psychological disturbances may be insufficiently captured. Therefore, the widely adopted TBI-specific HRQOL questionnaire QOLIBRI [82–84] will also be used as an important secondary outcome. Furthermore, the EuroQol-5D-5L questionnaire will be used for the economic evaluation [85]. Cognitive functioning will be assessed by the Montreal Cognitive Assessment (MOCA) [86]. The four questionnaires are briefly described:

##### GOS-E

The Glasgow Outcome Scale (Extended) is (together with its precursor GOS) the most commonly used global outcome measure in TBI research.

##### QOLIBRI

The Quality of Life after Brain Injury is the first TBI disease-specific quality-of-life outcome tool that is cross-culturally developed and validated in large populations.

##### EQ-5D-5L

EuroQol-5D-5L is a 5-dimensional generic instrument assessing health-related quality of life and health status and generates an index of health for use in economic evaluations.

##### MOCA

The Montreal Cognitive Assessment is a widely used questionnaire assessing 8 domains of cognitive functioning. As the MOCA cannot be completed per telephone, it will only be performed during the live visits up to 12 months.

All secondary outcomes are listed below.

- Functional outcome as expressed on the GOS-E besides the one year measurement (this includes mortality)
- Disease-specific quality of life as expressed on the QOLIBRI
- Health-related quality of life as expressed on the EuroQol-5D-5L
- Cognitive functioning as expressed on the MOCA
- Direct and indirect costs
- Duration of hospital stay
- Time from event to surgery
- Discharge locations
- Complications (during hospital stay)
- Secondary surgery in both groups

#### Other study parameters

With regard to the second research question, the investigators aim to identify subgroups of patients who will benefit substantially from one of the proposed treatments. It should be noted that these subgroup analyses are explorative in nature as this trial has been powered based on the primary effect estimate.

The following subgroups will be investigated:

1. Elderly with accompanying cerebral contusions visible on first CT versus isolated ASDHs
2. “Younger elderly” (aged 65–80 years) versus “older elderly” octogenarians (aged > 80 years)
3. Elderly receiving specific oral anticoagulants or antiplatelets versus elderly not receiving such medication*
4. Pre-trauma premorbid functionally independent of minimally dependent elderly (FIM score 4–7) versus functionally dependent elderly (FIM score 1–3) [69, 81]
5. Elderly with severe comorbidities (ASA III-V) versus elderly with mild or no comorbidities (ASA I/II) [82, 83]
6. Male versus female
7. Surgical technique: basic hematoma removal by craniotomy versus add-on decompression
8. No ICP monitoring versus ICP guided treatment taking into account cerebral perfusion pressure
9. Subgroups stratified on (automatically generated) measurements of volumes and ratio of volumes of important intracranial traumatic findings on non-contrast CT in the acute phase

*A subdivision will be made between anticoagulants (e.g., coumarin derivatives/heparin/DOACs) and antiplatelets (e.g., aspirin/clopidogrel/dipyridamole).

### Participant timeline {13}

**Fig. 4:**
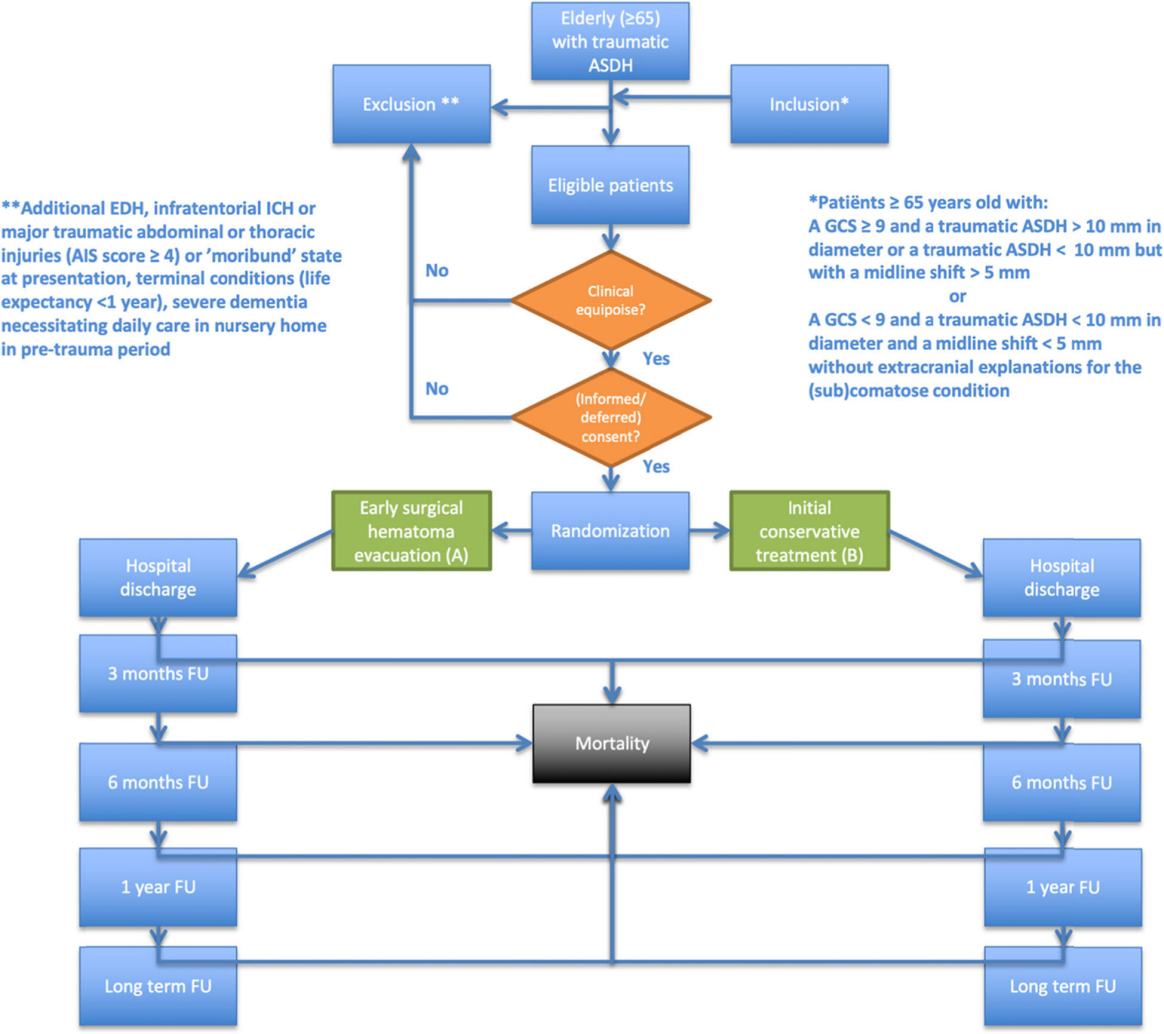
RESET-ASDH study design flowchart

Eligible patients will be randomized in one of two groups (early surgery or initial conservative management) directly after the initial CT scan and informed or deferred consent. This will mostly occur after arrival at one of the participating neurosurgical centers but may also take place in affiliated hospitals prior to transfer depending on the local agreements and working methods. Several questionnaires will be obtained by visiting participants at discharge, 3 months, 6 months, and 1 year. Long-term follow-up will take place yearly via telephone, postal questionnaires, or digitally for up to 5 years. The design of the trial is depicted in Fig. 4 and Table 3 describes the follow-up evaluation.

**Table 3:** Follow-up valuation

	Patient visit by research nurse				Telephone/postal			
Questionnaires	Discharge	3 months	6 months	1 year	2 years	3 years	4 years	5 years
Demographics	X							
GOS-E The Glasgow Outcome Scale (Extended) is (together with its precursor GOS) the most commonly used global outcome measure in TBI research	X	X	X	X^a^	X	X	X	X
QOLIBRI The Quality of Life after Brain Injury is the first TBI disease-specific quality-of-life outcome tool that is cross-culturally developed and validated in large populations		X	X	X	X	X	X	X
EQ-5D-5L EuroQol-5D-5L is a 5-dimensional generic instrument assessing health-related quality of life and health status and generates an index of health for use in economic evaluations		X	X	X	X	X	X	X
MOCA The Montreal Cognitive Assessment is a widely used questionnaire assessing 8 domains of cognitive functioning. As the MOCA cannot be completed per telephone, it will only be performed during the live visits up to 12 months		X	X	X				
Health economics Intramural care costs, patient costs and productivity loss of both patient and family will be determined through tailored patient questionnaires containing the relevant aspects of the iMCQ, iPCQ and iVIC questionnaires		X	X	X				X

^a^Primary outcome

### Sample size {14}

The sample size of 300 patients was based on an ordinal analysis method of the primary outcome. It was calculated analytically using PASS software version 11 and achieves a 90% power to detect a change in the log odds ratio of 0.69 with a 0.05 (two-sided) significance level, allowing for a loss of follow-up of 8%. Calculations were based on the expected percentage distribution of conservatively treated patients over the GOS-E categories 1–8 (15, 10, 10, 10, 10, 10, 15, 20) with category 1 and 2 combined and an estimated (adjusted) proportional odds ratio of 2.0 representing the targeted treatment effect that is considered clinically relevant in the management of elderly with a traumatic ASDH. Table 4 shows the corresponding distribution of patients over the GOS-E outcome categories. The rationale for combining GOS-E categories 1 and 2 in the statistical analysis is the opinion of the researchers that a potentially large shift of patients from category 1 (death) to category 2 (vegetative state) should not result in a positive trial as this cannot be considered a beneficial or positive effect of surgery.

**Table 4:** Estimated shift over GOS-E outcome categories

GOS-E	1&2	3	4	5	6	7	8
Patient’s condition	Dead and vegetative state	Lower severe disability	Upper severe disability	Lower moderate disability	Upper moderate disability	Lower good recovery	Upper good recovery
Initial conservative treatment (%)	25	10	10	10	10	15	20
Early surgery (%)	14	7	8	9	10	19	33

### Recruitment {15}

Ultimately, 300 patients will be included in the study. Yearly, a total of approximately 460 elderly patients are expected to present with a traumatic ASDH in all participating research centers combined. ASDH patients present to one of the participating hospitals during the inclusion period. From previous experience with CENTER-TBI and Net-Qure, it is estimated that approximately 25% of all consecutive ASDH patients in the centers will meet 1 or more exclusion criteria and 10% will refuse participation (with probably little higher refusal rates in large urban hospitals based on previous big-city experiences of the investigators). Based on the previously mentioned STITCH trial and clinical experience of the investigators, it is estimated that “clinical equipoise” will be present in approximately 85% of cases. Thus, the investigators estimate that approximately 50% of screened patients will actually get randomized. To achieve an inclusion of 300 patients, a 2-year recruitment period is estimated. A 32% margin for unforeseen issues has been taken into account in the calculation of the inclusion period. Importantly, neurosurgeons in all participating centers will be extensively tutored on location to become more familiar with the concept of “clinical equipoise” during planned visits by the PI and research team before start of the trial in order to optimize patient recruitment.

## Assignment of interventions: allocation

### Sequence generation {16a}

Randomization will take place with a 1:1 allocation ratio via a web-based randomization program by the treating clinician or including researcher and will be stratified by center and blocked with alternating block sizes of 2, 4, and 6. The randomization process will be recorded by the web-based randomization system. A 24-h randomization service will be backed by 24-h availability of the research team, who will also be able to advise on patient eligibility. In case of web-related problems, the treating clinician or including researcher will undertake randomization instead by manually opening a prefabricated sealed envelope containing a category.

### Concealment mechanism {16b}

A web-based randomization program will be used. In case of web-related problems, the treating clinician or including researcher will undertake randomization instead by manually opening a prefabricated sealed envelope containing a category. See also item 16a.

### Implementation {16c}

The treating clinician or including researcher will perform randomization via a web-based randomization program. See also item 16a.

## Assignment of interventions: Blinding

### Who will be blinded {17a}

It is not possible to blind either patients or treating neurosurgeons to whether or not the patient receives surgical hematoma evacuation. For organizational and ethical purposes, it is also not possible to blind the outcome evaluators, being the research nurses. The PhD students analyzing the data will however be blinded for the allocated treatment arm by means of a database that does not reveal the study group assignment.

### Procedure for unblinding if needed {17b}

This is not applicable; no unblinding will be necessary for the PhD students analyzing the data.

## Data collection and management

### Plans for assessment and collection of outcomes {18a}

#### Handling and storage of data and documents

After randomization, clinical care and medical documentation will take place according to local site protocol. Dedicated research nurses from Leiden/Leuven will collect all relevant data, such as pre-hospital, clinical, and imaging data. Data collection is done in standardized electronic databases, based on the “common data elements” for TBI and web-based data collection protocol [87, 88].

### Plans to promote participant retention and complete follow-up {18b}

Experienced research nurses for Leiden/Leuven will collect all relevant data, such as pre-hospital, clinical, and imaging data. In case of no response, attempts will be made to contact patients via their legal representative and/or general practitioner. If follow-up data from a patient is missing, values will be imputed based on other available follow-up data from that patient. Only patients without any follow-up data (including the 3-month assessment) will be considered “lost to follow-up”. This includes patients whose (deferred) consent is withdrawn before the 3-month follow-up assessment. Patients who have died soon after randomization will be included in the analysis as “death” is one of the outcome categories in the primary outcome measurement (GOS-E). Patients who were wrongfully included due to mistakes in the evaluation of objective entry criteria such as age (eligibility violations) will be considered “non-eligible” in the second instance. The amount of patients “lost to follow-up” or “non-eligible” in the second instance is estimated to be a very small proportion of all study patients (< 5%).

### Data management {19}

All relevant clinical data will be entered into electronic Case Report Forms (eCRFs). Data storage and backup will be managed by the CASTOR data management platform.

#### Integration with analytic platforms

The RESET-ASDH dataset will be registered on the DANS/easy archiving and networking service. The Dublin Core generic metadata scheme will be used for description of the data collection. Together with the CASTOR systems, the International Neuroinformatics Coordinating Facility (INCF) will ensure that data standards are established for the data model, e.g., conformity of field formats, field codes, and names to ensure consistency across all datasets. INCF will also be responsible for importing cleaned datasets to other analytic platforms as determined by the coordinating researchers.

### Confidentiality {27}

Data will be handled confidentially and coded in compliance with the European Union General Data Protection Regulation (GDPR) (In Dutch and Flemish: Algemene Verordening Gegevensbescherming). The CASTOR encryption module will be used to encrypt information capable of identifying individuals. The encryption code will only be available to selected members of the research team as well as independent data monitors and the Health and Youth Care Inspectorate (IGJ). All analyses will be performed on de-identified pseudonymized coded data, for which explicit permission is given in the patient informed consent form. A subject identification code list will be used to link the data to the subject when needed for data collection. Once assigned, the number will not be reused if the patient is excluded. After completion of the study, the key file will be archived in the hospital’s study documentation on a protected location on the network hard drive for 15 years in accordance with article 17 of the EU GCP directive. Patients or their legal representatives can withdraw their permission for data collection and storage at any time without consequences for the medical treatment. Any planned follow-up study visits will subsequently be canceled. Data that has already been collected before withdrawal of permission will be used for the analyses, unless specifically declined. A Data Protection Officer will be installed for the study to answer potential questions regarding processing of personal data and the involved legal aspects. For general information about data processing, either the Leiden University Medical Center (LUMC) or the Dutch Data Protection Authority can also be contacted.

### Plans for collection, laboratory evaluation, and storage of biological specimens for genetic or molecular analysis in this trial/future use {33}

This is not applicable; no biological specimens are collected.

## Statistical methods

### Statistical methods for primary and secondary outcomes {20a}

The primary outcome in this study will be the GOS-E at 1 year. Traditionally, such outcome scales are analyzed by dichotomizing the ordinal scale into a binary scale by defining outcome as “unfavorable” or “favorable” and calculating an odds ratio. However, many patients will not have a realistic opportunity to cross the threshold between “unfavorable” and “favorable” and will therefore not contribute data to the analysis. The crude odds ratio is thus not a meaningful effect measure for a large number of patients and discards much relevant information, reducing both the clinical relevance of the results and the statistical efficiency of the analysis. Therefore, the researchers consider it more appropriate to quantify effects across the full range of the GOS-E. In line with the IMPACT recommendations (NIH-funded International Mission for Prognosis and Analysis of Clinical Trials in TBI project), the investigators plan to analyze the primary outcome by using a proportional odds regression with covariate adjustment for age, GCS, and pupillary reactivity to adjust for baseline imbalances and to optimize statistical efficiency. Ordinal methods have been shown to increase statistical power substantially compared to traditional dichotomous analyses, equivalent to allowing a reduction of over 40% in the sample size without loss of statistical power. The primary effect estimate will be the adjusted proportional odds ratio with 95% confidence interval for the shift in the direction of a better outcome on the GOS-E. Ordinal logistic regression analysis is similar to logistic regression analysis, except for the fact that it estimates multiple odds ratios instead of one. The number of odds ratios is equivalent to the number of categories minus one. The final estimated effect size is a pooled estimate of the common odds ratio. The ordinal regression model assumes that the odds ratio for each potential cut of the GOS-E is constant no matter which cut-off point is taken (proportional odds assumption). Although the common odds ratio is formally only valid if the proportional odds assumption is met, the common odds ratio can be interpreted as a summary measure of treatment effect, even if the odds ratios differ by cut-off [89, 90].

In this study, the common odds ratio can be interpreted as the average shift over the GOS-E scale at 1 year caused by early neurosurgical intervention compared to non-operative management [90].

The investigators hold the opinion that a potential large shift of patients from GOS-E category 1 (death) to 2 (vegetative state) should not be considered a beneficial effect of surgery and should not result in a positive trial. Therefore, these categories will be combined in the final statistical analysis. As this trial is pragmatic, results will be analyzed according to an intention-to-treat protocol. Predefined subgroups for exploratory analyses are described below. The secondary outcomes will be analyzed using the appropriate tests. A *p*-value of less than 0.05 will be used to indicate statistical significance. For all analyses, commercially available statistical software like SPSS or R will be used.

#### Primary study parameter

The average shift over the GOS-E scale caused by early neurosurgical intervention compared to conservative treatment will be calculated using the appropriate tests. The primary analysis set for the primary endpoint is the intention-to-treat (ITT) population. This population must comply with the inclusion criteria and provide informed consent or be included under deferred consent, irrespective of adherence to the allocated treatment.

Our sample size allows us to detect a shift over the GOS-E (see Table 4) from the estimated distribution of conservatively treated patients conform a proportional odds ratio of 2.0 with a power of 90% and a 0.05 significance level (two-sided).

#### Secondary study parameters

For all secondary study parameters, subjects in both arms of the study will be compared using the appropriate tests, based on the ITT population. Graphic data displays may also be used to summarize the data. Statistical analyses may include logistic and linear regression models, Fisher’s exact tests, or chi-square tests and Student’s *t* tests or Mann-Whitney *U* tests (depending on normality of data). The interaction of different operative techniques with treatment arm outcome will be addressed in our subgroup analysis by a Cox Proportional Hazard model with the “day of discharge from the rehabilitation center, nursing, or hospital facility to the own home” as an endpoint/event, which is considered a relevant endpoint from both a QOL and a health-economic perspective. Importantly, analyses of subgroups representing dichotomized continuous variables may also be performed by an interaction-effect analysis.

### Interim analyses {21b}

This is not applicable; no interim analyses will be performed.

### Methods for additional analyses (e.g., subgroup analyses) {20b}

A cost-effectiveness analysis (CEA) and a cost-utility analysis (CUA) will be conducted to identify the costs per quality-adjusted life year (QALY) gained for both the surgically treated and the conservatively managed group [91].

#### Economic evaluation

An economic evaluation will be performed in accordance with the Dutch and Belgium guidelines for economic evaluations [92, 93]. It will be performed from a healthcare and societal perspective and will be estimated for a short-term (1 year) and long-term (5 years) period. A cost-effectiveness analysis (CEA; with GOS-E at 1 year as outcome) and a cost-utility analysis (CUA; with QALY as outcome) will be performed. With a decision model, the effectiveness in quality-adjusted life years (QALYs) [91], the costs, and the cost-effectiveness ratios (Euros/QALY) will be calculated for the long-term, using data from this study in combination with literature data.

#### Cost analysis

The cost analysis from the healthcare perspective will include direct healthcare costs, induced (indirect) healthcare costs (events, admissions, follow-up visits, follow-up procedures), and direct non-healthcare costs (patient time costs, out-of-pocket, and travel expenses). Resource utilization will be determined through documentation in the CRF. Hospital acute care costs will be calculated using reference prices as described in the national guidelines for healthcare costs research [92, 93]. The costs of post-discharge long-term care and rehabilitation care will be calculated by a similar method. Indirect costs as well as other direct non-medical costs will be obtained from patients or caregivers by questionnaires. Intramural care costs, patient costs and productivity loss of both patient and family will be determined through tailored patient questionnaires containing the relevant aspects of the iMCQ, iPCQ, and iVIC questionnaires. These questionnaires will be conducted by live visits or telephone interviews at 3 months, 6 months, 1 year, and 5 years follow-up. Costs of the interventions will be determined with extensive cost analysis enumerating costs of the equipment, personnel, materials, administration, and overhead. In Belgium, the national registry of intramural care will be used to validate parts of our questionnaire regarding healthcare use. Costs of complications and events during follow-up will be estimated. Importantly, previously neglected variables in the elderly population like familial childcare, retirement spending habits, volunteer work, or loss of productivity of caregiving family members will also be considered in the cost analysis.

#### Cost-effectiveness and cost-utility analysis

An incremental cost-effectiveness ratio (ICER) will be calculated comparing the costs and effects between the surgical and conservative treatment group. A CUA will be performed for which the QALY, calculated based on the EQ-5D-5L summary score, is the outcome measure. For the CEA, the primary effect measure will be the GOS-E. The costs and effects will be discounted. A 1-way, 2-way, and probabilistic sensitivity analysis will be performed to determine the effect of uncertainty in all input parameters.

### Methods in analysis to handle protocol non-adherence and any statistical methods to handle missing data {20c}

Analyses will be performed on the Full Analysis Set, which is defined by the ICH E9 guidelines “statistical principles for clinical trials” as “The set of subjects that is as close as possible to the ideal implied by the intention-to-treat principle. It is derived from the set of all randomized subjects by minimal and justified elimination of subjects” [94]*.* This means participants will be analyzed according to an intention-to-treat design, which involves analyzing patients according to the treatment arm to which they were initially randomized, including patients receiving delayed neurosurgical intervention after initial randomization to the non-operative arm. If follow-up data from a patient is missing, values will be imputed based on other available follow-up data from that patient. Only patients without any follow-up data (including the 3-month assessment) will be considered “lost to follow-up”. This includes patients whose (deferred) consent is withdrawn before the 3-month follow-up assessment. Patients who have died soon after randomization will be included in the analysis as “death” is one of the outcome categories in the primary outcome measurement (GOS-E). Patients who were wrongfully included due to mistakes in the evaluation of objective entry criteria such as age (eligibility violations) will be considered “non-eligible” in the second instance. The amount of patients “lost to follow-up” or “non-eligible” in the second instance is estimated to be a very small proportion of all study patients (< 5%).

### Plans to give access to the full protocol, participant-level data, and statistical code {31c}

The full protocol will be publicly accessible as published. Access to the patient-level dataset and statistical code can be provided in consultation with the principal and coordinating investigators. On completion of the trial, and after publication of the primary manuscript, data requests can be submitted to the researchers at the Leiden University Medical Center, department of neurosurgery.

## Oversight and monitoring

### Composition of the coordinating center and trial steering committee {5d}

The Leiden University Medical Center (LUMC), department of neurosurgery will serve as sponsor. The University Hospital Leuven (UZ Leuven), department of neurosurgery will serve as national coordinating center. The trial includes multiple participating centers spread over The Netherlands and Belgium. Both the sponsor and national coordinator will function as (co)chairs of the management team (MT). The management team consists of all clinical principal investigators and methodological specialists. The MT is responsible for the full cycle of data completion, analyses, and publications as well as financial expenditures and its accounting. A project team financial manager (LUMC) will monitor costs and personal output. Each PI represents a participating center. The PIs and their research group are responsible for the screening, intake, inclusion, and follow-up until discharge of patients. Further follow-up will be coordinated centrally by the sponsor and Belgian coordinating center. Per location, the PI has delegated responsibility of local ethical operational procedures and data completion. Also, they are responsible for the presence of a well-trained research team and properly utilized facilities. Data quality and completeness will regularly be checked by a data curation task force, supported by the epidemiological and statistical research members, being experienced TBI researchers in Leiden, Leuven, and Rotterdam. Independent researchers, all specialists in TBI care, will be involved in controlling on good clinical practice. The sponsor will organize proper study monitoring. Adverse events will be centrally reported and centrally analyzed. An independent medical doctor with clinical research expertise has been installed. The sponsor (LUMC) and Belgian coordinating center (UZ Leuven) hold monthly digital meetings in which the status of the trial and pending issues are discussed. In addition, a monthly meeting with ZonMw/KCE is held to discuss recruitment issues.

### Composition of the data monitoring committee, its role and reporting structure {21a}

#### Clinical monitoring

Data collection for each timepoint must be completed accurately and to schedule. Data monitoring will utilize both online monitoring of web entry forms and source data verification at the site level to optimize efficiencies and reduce data discrepancies. Monitoring in all sites in the Netherlands will be executed by (internal) LUMC monitors according to the monitoring plan. Similarly, monitoring in all sites in Belgium will be executed by monitors from the UZ Leuven. During these monitoring contacts, some subject records and eCRFs will receive a targeted review that may include items such as informed consent, eligibility, inclusion/exclusion criteria, scoring standard, and pseudonymization of data. Monitoring reports will be completed after every visit. Furthermore, the coordinating investigator will also conduct site visits every 6 months in all Dutch participating centers to assess protocol compliance; discuss enrolment practices, inclusion rates, and data collection; and deliver findings to the local researchers. A representative from the Belgian coordinating center will conduct similar half-yearly site visits in all the Belgian participating centers. Cross visits from the Dutch study staff/coordinating investigator are also planned once every 2 years per center in Belgium. The study staff and coordinating investigator will review the results of all monitoring visits and regularly scheduled data checks to identify trends and problems, and will share these with all research partners on a regular basis.

### Adverse event reporting and harms {22}

#### Temporary halt for reasons of subject safety

In accordance to section 10, subsection 4, of the WMO, the sponsor will suspend the study if there is sufficient ground that continuation of the study will jeopardize subject health or safety. The sponsor will notify the accredited METC without undue delay of a temporary halt including the reason for such an action. The study will be suspended pending a further positive decision by the METC. The investigator will take care that all subjects are kept informed.

#### Adverse events (AEs)

Adverse events are defined as any undesirable experience occurring to a subject during the study, whether or not considered related to one of the treatments (surgical or conservative). Given the fragility of the study patients and their susceptibility for AEs, these will not be recorded as this would involve a disproportionate workload without adding value, which is unwanted in this pragmatic study. Serious adverse events (SAEs), however, will be recorded as described below.

#### Serious adverse events (SAEs)

A serious adverse event is any untoward medical occurrence or effect that

- Results in death;
- Is life threatening (at the time of the event);
- Requires hospitalization or prolongation of existing inpatients’ hospitalization;
- Results in persistent or significant disability or incapacity;
- Any other important medical event that did not result in any of the outcomes listed above due to medical or surgical intervention but could have been based upon appropriate judgment by the investigator.
  An elective hospital admission will not be considered as a serious adverse event.

#### Annual safety report

As this study compares two existing treatments which are both considered standard of care, there is no legal obligation to report SAEs under the WMO. However, the sponsor shall provide the accredited METC in both Belgium and the Netherlands as well as the subsidizing parties twice a year during the first 3 years of the clinical trial with a progress report including a line listing of all SAEs that have occurred over this period and a report of the subject’s safety. These half-yearly reports will also include detailed information about the overall study status and recruitment status.

#### Follow-up of adverse events

All reported SAEs will be followed during the scheduled follow-up moments of this study until they have abated or until a stable situation has been reached. Depending on the event, follow-up may require additional tests or medical procedures as indicated, and/or referral to the general physician or a medical specialist.

#### Data Safety Monitoring Board (DSMB) / Safety Committee

There will be no Safety Committee installed for this study, because no new treatment is introduced. This study simply compares the effect of two generally accepted treatment modalities (early surgery versus initial conservative management) on functional outcome and will therefore not introduce any additional risks to the included patients compared to the existing risks outside of this study.

#### Premature termination of the study

Failure to recruit more than 25 patients during the first 6–9 months of the study may result in termination of the study, since this will make it unlikely that the intended sample size of 300 patients will be reached. Besides that, no pre-planned criteria for premature termination of the study are defined, as no interim efficacy analyses are planned and no experimental procedures are tested. In the event of premature termination of the study, outcome data for all included patients will be collected.

### Frequency and plans for auditing trial conduct {23}

#### Automated data integrity monitoring

All clinical data will be entered into electronic Case Report Forms (eCRFs) and managed by the CASTOR data management platform. As data is entered into each form, the system will run data validation checks that include conditionally required data, validation across fields, and validation requirements based on subject type. If any validation check fails, the user is alerted immediately that the data does not meet quality assessment criteria and the issue can be addressed and corrected at that point. If a data element fails a validation check, yet the value entered is correct, the user can enter an exception to the problem and provide a notation as to why the out-of-range data is actually correct. Data validation checks include:

- *Date/time value checks*: all dates and times entered into the database are checked to ensure that events recorded are accurate and in sequence.
- *Range value checks*: all numeric, non-date fields have range values specified to minimize data entry errors.
- *Selection lists*: all categorical data fields have predetermined drop-down lists, check boxes, or resettable radio buttons instead of free text to ensure accuracy.
- *Logic checks*: data fields from different sections of the eCRF will be compared to pass logical integrity.
- *Required fields*: the eCRF will be programmed to require input into fields when appropriate to minimize missing information.
- *Score calculation*: will be performed and programmed into eCRFs for tests and measures with numerical score summations or norming to avoid mathematical errors by the examiner. All automated scoring computations will be fully documented and validated by CASTOR and the Clinical Core, and must pass User Acceptance Testing.
- *Electronic data audits*: will be automated in the CASTOR database through a series of predetermined queries against the study database at regular intervals. These queries will be designed for the study staff to monitor data quality and completeness and identify protocol variations/deviations/violations.
- *Data audits against source documents*, where available, will be conducted in approximately 10% of subjects.

All investigators and designated study personnel will have unique and confidential password access to the CASTOR database. All access to the database and to study data will be logged in an audit trail and monitored. Any indication of inappropriate access will be reported immediately to the study coordinators. Investigators will have access to their data at any time. The database system will also provide checks for form completion based on the subject type. Validation rules will establish when forms for a particular subject should be entered, and any missing forms can be tracked by the study site and study management immediate follow-up. Once subject forms are marked complete, a dataset for sharing can be created. The CASTOR platform stores the exact dataset that is shared for future reference and also tracks information about when the data was shared and the dataset recipient. Due dates for eCRF completion windows are set by the study management. The CASTOR system will automatically generate reminders to complete eCRFs for enrolled patients. Reports of enrolment, timeliness of eCRF completion, and error correction will be monitored and adjudicated by independent data monitors.

### Plans for communicating important protocol amendments to relevant parties (e.g., trial participants, ethical committees) {25}

Amendments are changes made to the research after a favorable opinion by the study management and accredited METC has been given. All amendments will be notified to the METC that gave a favorable opinion. The study staff will submit a summary of the progress of the trial to the funding agencies and the accredited METC every 6 months. Information will be provided on the date of inclusion of the first subject, numbers of subjects included, and numbers of subjects that have completed the trial, a line listing of serious adverse events, other problems, and amendments. Patients were involved in the form of a “patient advisory panel” consisting of “expert patients” and their caregivers from The Netherlands, Flanders, and French-speaking Belgium who helped in the trial design and selection of appropriate outcome measures. The panel will be informed about relevant study developments and outcomes and will be encouraged to participate in the trial progress discussions.

#### Temporary halt and (prematurely) end of study report

The investigator/sponsor will notify the accredited METC of the end of the study within a period of 8 weeks. The end of the study is defined as the last patient’s last visit. The sponsor will notify the METC immediately of a temporary halt of the study, including the reason of such an action. In case the study is ended prematurely, the sponsor will notify the accredited METC within 15 days, including the reasons for the premature termination. Within 1 year after the end of the study, the investigator/sponsor will submit a final study report with the results of the study, including any publications/abstracts of the study, to the accredited METC.

### Dissemination plans {31a}

#### Public disclosure and publication policy

The first publication in respect of the findings resulting from the clinical study and its primary endpoint shall emanate from the coordinating investigator, principal investigators, and other involved investigators in peer-reviewed journals and shall be presented at national and international meetings. The funding agencies (ZonMw and KCE) are also entitled to publish details of the selection process, the research objectives, plan, and costs of the clinical study.

#### Implementation of study results

The results of this trial will provide an evidence-based medical strategy that is likely to be adopted and implemented, since high-quality evidence is currently clearly lacking. High-quality high-impact peer-reviewed publications as well as social media attention, podium presentations and activities of the two principal investigators together with the local coordinators per center in the International Guideline Committee for Neurotrauma of the WFNS, EANS, INTS, and WHO will guarantee exposure and accelerate the worldwide implementation. The Dutch PI is a member of the WFNS NeuroTrauma Committee and linking pin to WHO and active in the global implementation of trauma guidelines and training. Study results could both lead to superiority of (cost-)effectiveness of early surgical treatment or conservative treatment in elderly patients with a traumatic ASDH. Depending on the results, the current BTF guidelines recommendations will either be supported by high-quality evidence or will have to be altered. Because participating departments of the Netherlands and Belgium have already been performing both interventions in their clinical practices and treatment variation is very common, both outcomes will directly influence daily clinical practice worldwide. An implementation expert will also be consulted to design a dissemination and implementation plan (DIP) tailored to the results of the study. A decision aid for patients, caregivers, and clinicians will be developed based on the study results, which will provide evidence-based information about the disease, the—surgical versus initially conservative—treatment options and their associated benefits and harms. The use of decision aids in complex surgical treatment decisions have previously shown to improve patient knowledge about the subject and lower decisional conflict for patients without raising their anxiety levels [95]. The researchers have contact with various patient organizations in The Netherlands and Belgium, which will be helpful in spreading information among future patients.

## Discussion

The surgical versus conservative treatment of elderly with a traumatic ASDH remains an important dilemma as it is often unclear which treatment leads to a better outcome for the patient. The limited literature on (surgical) treatment of t-ASDHs is inconclusive regarding a preferred treatment strategy, partly because of common (selection) biases leading to self-fulfilling prophesies and skewed results [10, 17, 43–49]. Indeed, establishing causality based on non-randomized data is often impossible because of confounding by indication. A recent study comparing treatment strategy on a center level rather than on a patient level to reduce confounding by indication showed that an aggressive surgical management strategy was associated with better outcome in an elderly population with t-ASDH [30]. A systematic review regarding functional outcome of surgically and conservatively treated acute subdural hematoma patients is currently in the making [96]. Few studies have specified the type of surgical intervention and if they did, they did not address the effectiveness of the procedure [25]. Specifically, the decision to pre-emptively perform a decompressive craniectomy (DC) after evacuating the ASDH in an attempt to prevent increased intracranial pressure (ICP) due to brain swelling after surgery is outweighed against the high morbidity and mortality of a DC, especially in the elderly population [50]. The choice for craniotomy (CR) or DC in patients with ASDH has been shown to vary considerably [29, 51]. A randomized trial investigating DC versus CR for patients with traumatic ASDHs has recently finished patient recruitment [52]. While this trial might provide valuable information regarding the preferred operative strategy, it does not answer the question whether to operate or not. This decision-making process is currently complicated by the absence of a firm evidence base for treatment choice, defined as clinical equipoise, leading to low guideline adherence and large treatment variation. Although clinical equipoise provides the foundation for a randomized investigation, it also constitutes a well-known challenge for patient recruitment in surgical RCTs as (neuro)surgeons are highly trained decision makers. Eventually, the trial results will provide an evidence-based medical strategy that either substantiates current BTF guidelines or provides a strong incentive to alter them. Implementation of the results will be facilitated by the widespread use of both interventions in all participating centers spanning a large geographical area. From a health-economic perspective, reducing the amount of early surgery in elderly ASDH patients would result in obvious (in-hospital) cost savings [97]. On the other hand, if surgery turns out to be more effective, an incremental cost analysis could still prove surgical treatment to be more cost-effective on the long term [60, 61]. In this respect, the results of the CEA and CUA will improve health-economic moral deliberations and pave the way to more cost-effective treatment of this rapidly increasing patient group in an economically challenged healthcare system. Most importantly, the trial results will aid in solving the current clinical and moral dilemma by providing sound evidence on which treatment strategy leads to a better outcome for elderly patients with a t-ASDH.

## Trial status

Patient recruitment has suffered delays due to the COVID-19 pandemic as the elderly population is a critical group for both diagnoses. During the different peaks of COVID-19 “waves” and the accompanied shortage of ICU beds, it was deemed unethical to randomize patients for a neurosurgical hematoma evacuation with accompanied ICU admission when they could potentially also be treated with an initial conservative management on the ward. Now that the pinnacle of the COVID-19 crisis seems to be behind us, patient recruitment is planned to start in March 2022.

## Data Availability

The final trial dataset can be accessed by third parties in consultation with the sponsor (LUMC) and Belgian coordinating center (UZ Leuven). The first publication in respect of the findings resulting from the clinical study and its primary endpoint shall emanate from the coordinating investigator, principal investigators, and other involved investigators in peer-reviewed journals and shall be presented at national and international meetings. The funding agencies (ZonMw and KCE) are also entitled to publish details of the selection process, the research objectives, plan, and costs of the clinical study. All relevant agreements are captured in a consortium agreement between the LUMC and UZ Leuven as well as in Clinical Trial Agreements (CTAs) with all participating centers.

## Abbreviations

AE: Adverse event
AIS: Abbreviated Injury Scale
ASDH: Acute subdural hematoma
BTF: Brain Trauma Foundation
CCMO: Central Committee on Research Involving Human Subjects; in Dutch: Centrale Commissie Mensgebonden Onderzoek
CER: Comparative Effectiveness Research
CENTER-TBI: Collaborative European NeuroTrauma Effectiveness Research in TBI
CPP: Cerebral perfusion pressure
CR: Craniotomy
CRF: Case Report Form
CSF: Cerebrospinal fluid C
CTA: Clinical Trial Agreement
CUA: Cost-utility analysis
DC: Decompressive craniectomy
DSMB: Data Safety Monitoring Board
eCRF: Electronic Case Report Form
EDH: Epidural hematoma
EU: European Union
EudraCT: European drug regulatory affairs Clinical Trials
GCP: Good Clinical Practice
GCS: Glasgow Coma Scale
GDPR: General Data Protection Regulation
GOS-E: Glasgow Outcome Scale – Extended
GUPI: Generated Unique Patient Identifier
HRQOL: Health-Related Quality Of Life
IC: Informed consent
ICH: Intracerebral hemorrhage
ICP: Intracranial pressure
IMP: Investigational Medicinal Product
ITT: Intention to treat
METC: Medical research ethics committee (MREC); in Dutch: medisch ethische toetsing commissie (METC)
Net-QuRe: Neurotraumatology Quality Registry
(S)AE: (Serious) adverse event
Sponsor: The sponsor is the party that commissions the organization or performance of the research, for example a pharmaceutical company, academic hospital, scientific organization or investigator. A party that provides funding for a study but does not commission it is not regarded as the sponsor, but referred to as a subsidizing party
SUSAR: Suspected Unexpected Serious Adverse Reaction
TBI: Traumatic brain injury
WMO: Medical Research Involving Human Subjects Act (in Dutch: Wet Medisch-wetenschappelijk Onderzoek met Mensen)
QALY: Quality-adjusted life year
